# Interaction between a Unique Minor Protein and a Major Capsid Protein of Bluetongue Virus Controls Virus Infectivity

**DOI:** 10.1128/JVI.01784-17

**Published:** 2018-01-17

**Authors:** Eiko Matsuo, Kiyoshi Yamazaki, Hiroki Tsuruta, Polly Roy

**Affiliations:** a Microbiology & Immunology, Division of Animal Science, Department of Bioresource Science, Graduate School of Agricultural Science, Kobe University, Kobe City, Japan; b Faculty of Infectious and Tropical Diseases, London School of Hygiene and Tropical Medicine, London, United Kingdom; c Research Unit for Future Creation & Innovation Creative Dojo, Graduate School of Engineering, Kobe University, Kobe City, Japan; d Office for Academic and Industrial Innovation, Center for Applied Structural Science, Kobe University, Kobe City, Japan; Loyola University Medical Center

**Keywords:** BTV, reverse genetics system, VP6 translocation

## Abstract

Among the Reoviridae family of double-stranded RNA viruses, only members of the Orbivirus genus possess a unique structural protein, termed VP6, within their particles. Bluetongue virus (BTV), an important livestock pathogen, is the prototype Orbivirus. BTV VP6 is an ATP-dependent RNA helicase, and it is indispensable for virus replication. In the study described in this report, we investigated how VP6 might be recruited to the virus capsid and whether the BTV structural protein VP3, which forms the internal layer of the virus capsid core, is involved in VP6 recruitment. We first demonstrated that VP6 interacts with VP3 and colocalizes with VP3 during capsid assembly. A series of VP6 mutants was then generated, and in combination with immunoprecipitation and size exclusion chromatographic analyses, we demonstrated that VP6 directly interacts with VP3 via a specific region of the C-terminal portion of VP6. Finally, using our reverse genetics system, mutant VP6 proteins were introduced into the BTV genome and interactions between VP6 and VP3 were shown in a live cell system. We demonstrate that BTV strains possessing a mutant VP6 are replication deficient in wild-type BSR cells and fail to recruit the viral replicase complex into the virus particle core. Taken together, these data suggest that the interaction between VP3 and VP6 could be important in the packaging of the viral genome and early stages of particle formation.

**IMPORTANCE** The orbivirus bluetongue virus (BTV) is the causative agent of bluetongue disease of livestock, often causing significant economic and agricultural impacts in the livestock industry. In the study described in this report, we identified the essential region and residues of the unique orbivirus capsid protein VP6 which are responsible for its interaction with other BTV proteins and its subsequent recruitment into the virus particle. The nature and mechanism of these interactions suggest that VP6 has a key role in packaging of the BTV genome into the virus particle. As such, this is a highly significant finding, as this new understanding of BTV assembly could be exploited to design novel vaccines and antivirals against bluetongue disease.

## INTRODUCTION

Bluetongue virus (BTV), a member of the Orbivirus genus of the Reoviridae family, is the etiological agent of bluetongue disease of livestock, which particularly affects sheep and cattle. BTV particles are organized into two capsids: an outer capsid composed of two proteins (VP2 and VP5) and an inner icosahedral capsid (core) composed of two major proteins, VP7 and VP3 (1). Within the core, the virus encapsidates the 10 double-stranded RNA (dsRNA) genome segments (segments S1 to S10) and two enzymatic proteins, the polymerase (VP1) and a capping enzyme (VP4). In addition to these two enzymatic proteins, which are common across the Reoviridae, BTV and other orbiviruses possess an additional unique minor protein, VP6, which is a 36-kDa structural protein that has been shown to be an essential component of BTV replication and to possess helicase activity (2–5). In addition to the seven structural proteins, the BTV genome also encodes at least four nonstructural (NS) proteins (NS1, NS2, NS3, and NS4) (6–8).

During cell entry, which occurs via clathrin-mediated endocytosis, the outer capsid of the double-capsid BTV particle is removed, releasing the inner core particle to the cytoplasm. Without further disassembly, the intact core particle, which includes the transcription complex (VP1, VP4, and VP6), initiates the transcription of each of the 10 genomic dsRNA segments and simultaneously extrudes the newly synthesized single-stranded RNA (ssRNA) transcripts into the cytoplasm (9, 10). These ssRNA transcripts then act as the templates for both viral protein synthesis and negative-strand viral RNA synthesis during dsRNA genome synthesis (1, 9). The abundant nonstructural protein NS2 initiates the formation of virus assembly factories, known as “virus inclusion bodies” (VIBs) (11, 12). The inner core protein VP3, as well as the enzymatic proteins VP1, VP4, and VP6, is recruited to the VIBs with the ssRNA transcripts (4, 13–16). Within the VIBs, the subcore particles (i.e., particles lacking VP7) are assembled (3, 4, 16–19). Our current data suggest that after subcore assembly, VP7 is added onto the VP3 layer to form the stable core particle (13). This is subsequently released from VIBs and acquires the two outer capsid proteins, VP2 and VP5, to form mature infectious virions prior to virus egress (20). However, the details of core assembly remain unclear.

Current evidence suggests that VP6 is a multifunctional protein with RNA-binding ability and ATP-binding and ATP hydrolysis activity, in addition to ATP-dependent RNA unwinding activity (5, 21). As such, VP6 may act as a helicase during transcription of genomic RNA segments. However, neither the location of VP6 in the virus core nor the interactions between VP6 and other structural proteins have yet been elucidated.

Recently, using BTV reverse genetics (RG)-based studies, it has been possible to demonstrate that NS2 and a primary replicase complex consisting of all four subcore proteins are required during the early stages of virus replication (2–4). Specifically, we have demonstrated that BTV strains engineered to lack VP6 are replication deficient in a non-VP6-complementing cell line (wild-type BSR [WT-BSR] cells), whereas they replicate efficiently in a BSR cell line that constitutively expresses a functional VP6 (BSR-VP6 cells) (2, 3). However, when progeny viruses of VP6-deficient viruses grown in BSR-VP6 cells are used to subsequently infect WT-BSR cells, a core-like particle comprising VP3 and VP7 but lacking the genomic dsRNA could be purified (4). Thus, VP6 seems to play a key role in virus replication and genome packaging.

To understand further the structure-function relationship of VP6 in relation to capsid assembly, we investigated the localization of VP6 in relation to the inner core protein VP3. Subsequently, a series of VP6 mutants was generated to investigate whether VP6 directly interacts with VP3 and, if so, which residues are involved in such interactions. By using protein expression systems in combination with immunoprecipitation and size exclusion chromatography, we identified the critical residues of VP6 that are responsible for interaction with VP3. Furthermore, using our BTV RG system, we demonstrated that the interaction of VP6 with VP3 is essential for virus replication and genome packaging in a live virus system.

## RESULTS

### The C-terminal region of VP6 is involved in the translocation of VP6 into VIBs.

The BTV structural protein VP6 is an essential component of the primary replication complex and is involved in assembly of functional core particles within VIBs (3, 4, 13–16). Although a previous report suggested that VP6 associates with NS2 (the major component of VIBs) (16), the regions and/or sequences of VP6 involved in its translocation to the VIBs and its interactions with other components of VIBs are unknown.

**FIG 1:**
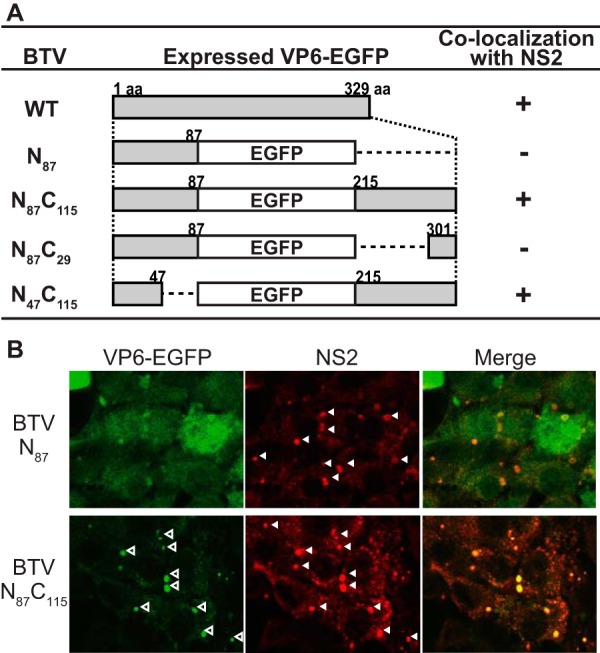
Localization of chimeric VP6-EGFP proteins in WT-BSR cells infected with various chimeric BTV strains. (A) Schematic representation of the changes introduced in BTV VP6. The name of the mutation is indicated on the left. Numbers in the middle column indicate amino acid (aa) positions in VP6, where EGFPs were fused with the N- and/or C-terminal region of VP6. +, colocalization of VP6-EGFP with NS2. (B) Colocalization of VP6 to which EGFP was fused at the C terminus with NS2. Either BTV/N_87_ (top) or BTV/N_87_C_115_ (bottom) was used to infect WT-BSR cells at an MOI of 1.0. At 24 h postinfection, the expression of EGFP and NS2 was observed using confocal microscopy. NS2 was detected using an anti-NS2 antibody produced in a guinea pig. Open and closed arrowheads, punctate structures of VP6 and VIBs, respectively.

To elucidate the regions of VP6 important for these interactions, we generated four VP6 proteins with truncations at the carboxy-terminal (C-terminal) region, as our previous data demonstrated that the amino-terminal (N-terminal) region (residues 34 to 92), which is highly flexible, is not essential for BTV replication in the cell culture system (22). All truncated regions of VP6 were replaced by enhanced green fluorescent protein (EGFP) to produce VP6 proteins fused with EGFP (VP6-EGFP proteins) (Fig. 1A), resulting in two constructs in which EGFP was fused to the VP6 protein and two in which EGFP was flanked by regions of VP6. The first, VP6/N_87_, contained the N-terminal 87 residues fused with EGFP by inserting a stop codon (TAG) at the end of the EGFP gene. The second, VP6/N_87_C_115_, was constructed with EGFP flanked by 87 N-terminal and 115 C-terminal residues. VP6/N_87_C_115_ was created by deleting the guanine of the stop codon at the end of the EGFP gene. The third mutant, VP6/N_87_C_29_, was constructed with EGFP flanked by 87 N-terminal and 29 C-terminal residues of VP6. The fourth mutant, VP6/N_47_C_115_, was constructed with 47 N-terminal and 115 C-terminal residues of VP6 fused at either end of EGFP, to confirm that the N-terminal region is unimportant for this function.

The four VP6-EGFP genes were introduced into the BTV genome using our RG system. Each BTV carrying a VP6-EGFP gene could be recovered only from BSR-VP6 cells and not from WT-BSR cells, indicating that each mutant could replicate only in the presence of wild-type (WT) VP6 (data not shown). While none of these four viruses could propagate in WT-BSR cells, each generated VP6-EGFP (data not shown). These data are consistent with our previous results, which demonstrated that the large truncation (amino acids 88 to 329) of VP6 perturbed virus replication in the absence of WT VP6 (2–4).

We next infected WT-BSR cells with each of the four BTV VP6-EGFP-expressing strains recovered from BSR-VP6 cells, and the expression and localization of VP6-EGFP in the WT cells were examined. Two VP6-EGFP proteins with intact C termini, VP6/N_87_C_115_ and VP6/N_47_C_115_, colocalized with NS2 in WT-BSR cells at 24 h postinfection (Fig. 1A and B). In contrast, the remaining two VP6-EGFP proteins lacking all or most of the C-terminal ends, VP6/N_87_ and VP6/N_87_C_29_, were detectable throughout the cytosol but were not colocalized with NS2 in WT-BSR cells (Fig. 1A and B, top). Together these data suggest that the C-terminal region of VP6, especially residues 215 to 301, might be essential for the translocation of VP6 to VIBs.

### The interaction between VP6 and NS2 occurs via the internal major capsid protein VP3.

**FIG 2:**
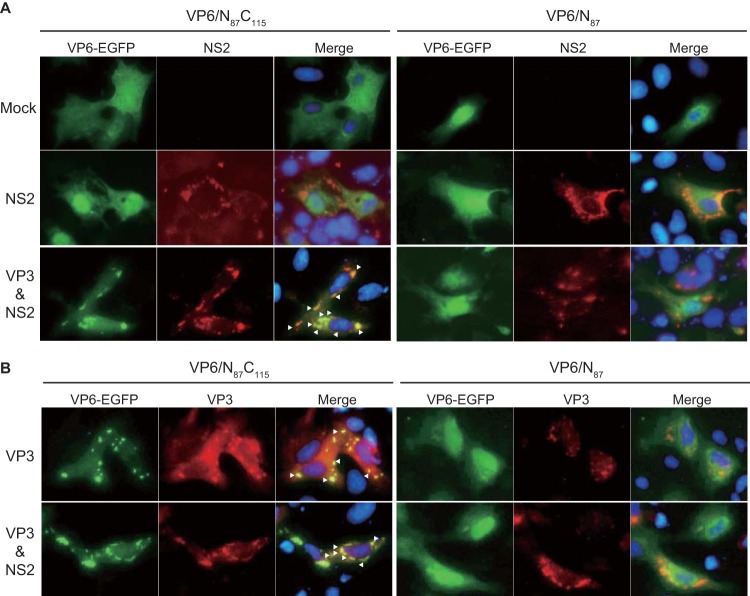
Translocation of VP6 mutants in the presence of VP3. (A) Colocalization of VP6 to which EGFP was fused at the C terminus with NS2 in the presence of VP3. Each of the mammalian expression plasmids, pCAG-PBTV10-VP6/N_87_C_115_ (left) and pCAG-PBTV10-VP6/N_87_ (right), was cotransfected with either pCAG-PBTV1-NS2 alone (middle) or pCAG-PBTV1-NS2 with pCAG-PBTV1-VP3 (bottom) into WT-BSR cells. As a control, pCAG-PBTV10-VP6/N_87_C_115_ and pCAG-PBTV10-VP6/N_87_ was each transfected alone into the cells (top). At 24 h posttransfection, the distributions of VP6-EGFP were observed using fluorescence microscopy. NS2 was detected using a guinea pig anti-NS2 antibody. Arrowheads, colocalization of VP6 with NS2. Nuclei were detected using DAPI. (B) Colocalization of VP6 to which EGFP was fused at the C terminus with VP3. Either VP6/N_87_C_115_ (left) or VP6/N_87_ (right) was coexpressed with VP3, using mammalian expression plasmids, in the absence (top) or presence of NS2 (bottom) in WT-BSR cells. The distributions of VP6-EGFP were observed using fluorescence microscopy. VP3 was detected using a mouse anti-VP3 antibody. Nuclei were detected using DAPI. Arrowheads, colocalization of VP6-EGFP and VP3.

To further define the role of the VP6 C-terminal region in the translocation of VP6 to VIBs, VP6-EGFP construct VP6/N_87_C_115_ (in which EGFP is fused to VP6) was coexpressed with NS2 in WT-BSR cells (Fig. 2A). In parallel, VP6-EGFP construct VP6/N_87_ (in which EGFP is not fused to VP6) was also coexpressed with NS2 in WT-BSR cells.

When VP6/N_87_C_115_ and VP6/N_87_ were expressed individually in WT-BSR cells, in which no other viral proteins were present, both were observed throughout the cytosol and in the nuclei (Fig. 2A, top). When VP6/N_87_C_115_ was coexpressed with NS2 in WT-BSR cells, VP6/N_87_C_115_ neither formed a typical punctate structure nor colocalized with NS2 (Fig. 2A, left, middle). In addition, coexpression of VP6/N_87_ with NS2 also did not alter its distribution in transfected WT-BSR cells (Fig. 2A, right, middle). These results suggest that the C-terminal region of VP6 does not directly interact with NS2, implying that the translocation of VP6 to VIBs is not dependent on NS2.

Since not only VP6 but also the other components of the core particles, including BTV RNAs, were visualized within the VIBs of BTV-infected cells (13–16), VP6 may be translocated to VIBs via other components of the core. To investigate this, plasmids expressing one of the core proteins, VP1, VP3, VP4, or VP7, alongside an NS2-expressing plasmid and a set of 10 T7 plasmid transcripts were cotransfected into WT-BSR cells with a plasmid expressing either VP6/N_87_C_115_ or VP6/N_87_. Only the coexpression of VP6/N_87_C_115_ with VP3 and NS2 clearly altered the distribution of VP6/N_87_C_115_, which colocalized with NS2 (Fig. 2A, left, bottom). The coexpression of VP6/N_87_ did not result in the colocalization of VP6/N_87_ with NS2 either in the absence or in the presence of VP3 (Fig. 2A, right, middle and bottom). None of the other core proteins or RNAs affected the distribution of VP6/N_87_C_115_ (data not shown). These data suggest that the C-terminal region of VP6 interacts with VP3 to facilitate the translocation of VP6 to VIBs.

**FIG 3:**
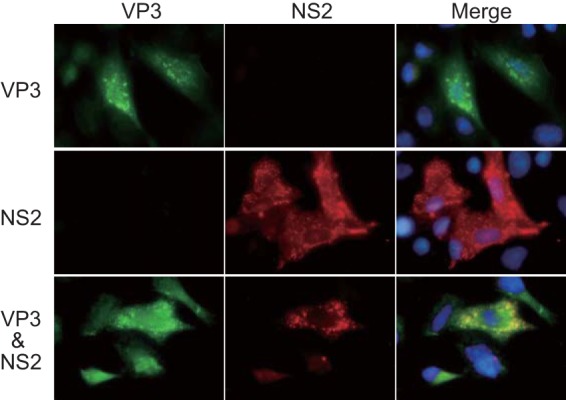
Colocalization of VP3 with NS2. VP3 was coexpressed with NS2 in WT-BSR cells (bottom). As a control, either VP3 (top) or NS2 (middle) was singly expressed in the cells. VP3 and NS2 were detected using a mouse anti-VP3 antibody and a guinea pig anti-NS2 antibody, respectively.

To confirm that the C-terminal region of VP6 interacts with VP3, we coexpressed VP3 with either VP6/N_87_C_115_ or VP6/N_87_ both in the presence and in the absence of NS2 (Fig. 2B). In WT-BSR cells, VP6/N_87_C_115_ colocalized with VP3 both in the presence and in the absence of NS2 (Fig. 2B, left). In contrast, the colocalization of VP6/N_87_ with VP3 was not observed, even when NS2 was present (Fig. 2B, right). Furthermore, when VP3 was expressed alone, it produced foci in WT-BSR cells (Fig. 3, top), and when it was expressed with NS2, VP3 colocalized with NS2 (Fig. 3, bottom). These results suggest that the C-terminal region of VP6 interacts with VP3 and that this VP6/VP3 interaction is likely to be involved in the recruitment of VP6 into VIBs.

### An 8-amino-acid motif in the C-terminal region of VP6 is responsible for direct interaction with VP3.

**FIG 4:**
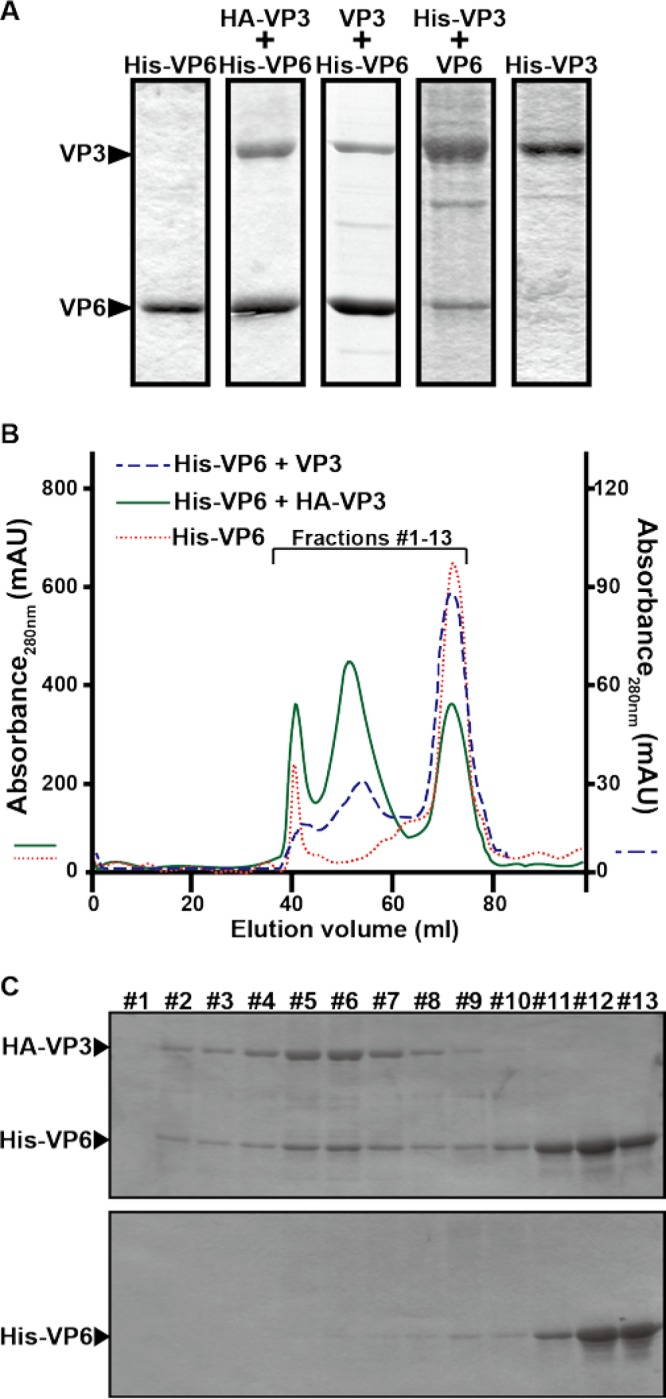
Interaction of VP6 with VP3. (A) Copurification of VP6 with VP3, each of which was expressed using a baculovirus expression system. His-tagged VP6 (His-VP6) was coexpressed with either HA-tagged VP3 (HA-VP3) or nontagged VP3 (VP3) in Sf9 cells. In parallel, His-tagged VP3 (VP3) was coexpressed with nontagged VP6 (VP6). As a control, either His-VP6 or His-VP3 was expressed. Proteins were purified with a His-Select nickel affinity gel. (B) Analysis of the VP6/VP3 complex by gel filtration chromatography. Two types of VP3/VP6 complexes, His-VP6/HA-VP3 (green solid line) and His-VP6/VP3 (blue dashed line), copurified using nickel affinity gels, were loaded onto an equilibrated HiPrep 16/60 Sephacryl S-300 HR gel filtration column and eluted with the same buffer at a flow rate of 0.5 ml/min. His-VP6 (red dotted line) was loaded as a control. The left vertical axis indicates the absorbance at 280 nm of His-VP6 and the His-VP6/HA-VP3 complex. The right vertical axis indicates the absorbance at 280 nm of the His-VP6/VP3 complex. mAU, milli-absorbance units. (C) Fractions of the His-VP6/HA-VP3 complex (fractions 1 to 13) were collected between elution volumes of 38 ml and 77 ml and analyzed using SDS-PAGE (top). As a control, the same fractions of His-VP6 were analyzed (bottom).

To further define the region of VP6 which interacts with VP3, recombinant baculoviruses expressing full-length His-tagged VP6 (His-VP6), hemagglutinin (HA)-tagged VP3 (HA-VP3), His-tagged VP3 (His-VP3), as well as untagged VP6 or VP3 were generated and used in coexpression, copurification, and biochemical analyses (Fig. 4).

In copurification analyses, where tagged and untagged VP6 and VP3 were expressed and complexes were purified using nickel affinity columns, we found that VP6 and VP3 interacted to form complexes (Fig. 4A). To confirm further the direct interaction of VP6 with VP3, the His-VP6/HA-VP3 complex was analyzed using size exclusion chromatography (Fig. 4B). In the elution profile of His-VP6 purified alone, two peaks were observed: one major peak corresponding to His-VP6 and a second minor peak, at the void fractions, likely to be a contaminated cellular protein (Fig. 4B, red dotted line). The elution profile of His-VP6 copurified with HA-VP3 showed the same two peaks as well as a third peak, which was likely a heterocomplex of His-VP6 and HA-VP3 (Fig. 4B, green solid line). The fractions eluted from the size exclusion columns were analyzed further by SDS-PAGE (Fig. 4C). The data confirmed that the first peaks had no VP6, while the second peaks were indeed VP6/VP3 complexes and the third peaks contained only VP6. Although a small amount of the complex was eluted with the untagged VP3 in comparison to the amount that eluted with the HA-VP3/His-VP6 complexes, the elution profiles were the same in both cases (Fig. 4B, blue dashed line).

**FIG 5:**
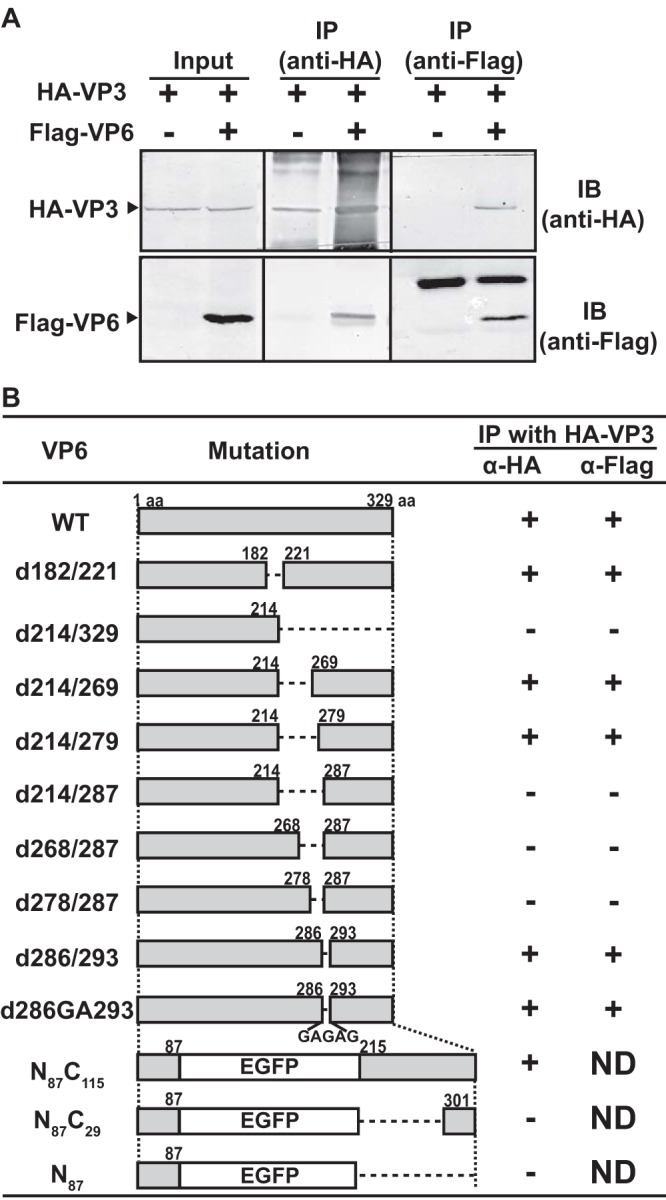
Direct interaction of VP6 with VP3 at a region between residues 279 and 286. (A) Coimmunoprecipitation of VP6 with VP3. HA-tagged VP3 (HA-VP3) was expressed in WT-BSR cells in the presence or absence of Flag-tagged VP6 (Flag-VP6). HA-VP3 and Flag-VP6 were immunoprecipitated using a mouse anti-HA MAb and a mouse anti-Flag MAb, respectively. Precipitated proteins were detected by immunoblotting using a rabbit anti-HA pAb and a rabbit anti-Flag pAb, respectively. (B) Schematic representation of modified VP6. Numbers in the middle column indicate amino acid positions in VP6 where changes were introduced. Note that no Flag tag was inserted at the N-terminal end of the three VP6 mutants N_87_C_115_, N_87_C_29_, and N_87_. +, coimmunoprecipitation; IP, immunoprecipitation; IB, immunoblotting; aa, amino acid; ND, not determined.

The direct interaction of VP6 with VP3 was further examined by coimmunoprecipitation, using an N-terminal Flag-tagged VP6 (Flag-VP6) and an N-terminal HA-tagged VP3 (HA-VP3). These were coexpressed in WT-BSR cells and immunoprecipitated using either anti-Flag antibody or anti-HA antibody (Fig. 5A). Flag-VP6 coprecipitated with HA-VP3 (as precipitated using an anti-HA antibody), and HA-VP3 coprecipitated with Flag-VP6 (as precipitated using an anti-Flag antibody), thus further demonstrating that VP6 directly interacts with VP3 (Fig. 5A).

To define the residues in the VP6 C terminus involved in the VP3 interaction, a series of Flag-tagged VP6 truncation mutants and three untagged chimeric VP6-EGFP mutants was tested for their binding to HA-VP3 by coimmunoprecipitation (Fig. 5B). Truncation of residues 279 to 286 ablated the ability of VP6 to coprecipitate VP3, suggesting that C-terminal residues 279 to 286 of VP6 may comprise the VP3-binding motif.

### VP6 binds VP3 via specific residues within its C-terminal region.

**FIG 6:**
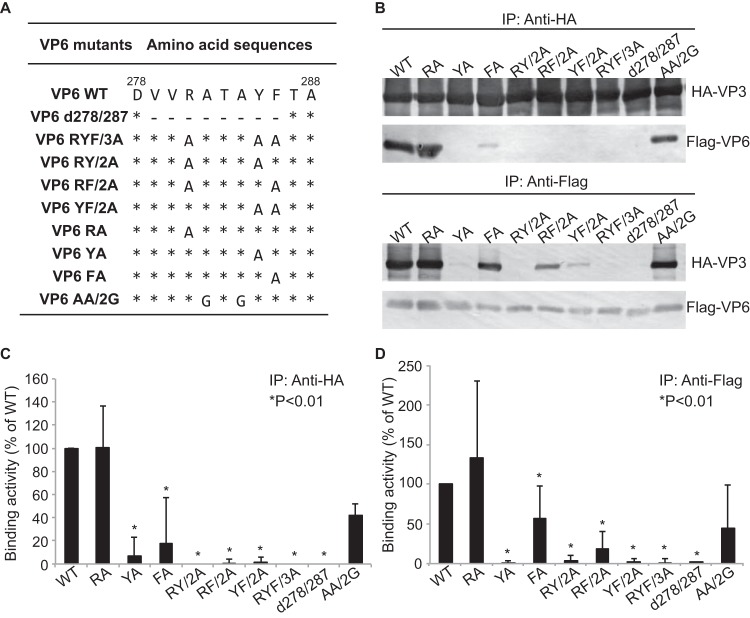
Mapping of the amino acid residues of VP6 essential for VP3 binding. (A) Schematic representation of the changes introduced into Flag-VP6. The name of the mutation is indicated on the left. Numbers indicate amino acid positions according to the VP6 amino acid sequence. The amino acid changes are also shown. Asterisks indicate no change, and dashes indicate deletions. (B) The interaction between HA-VP3 and a series of Flag-VP6 mutants was analyzed by immunoprecipitation using either anti-HA MAb (top) or anti-Flag MAb (bottom). (C) The relative densities of Flag-VP6 mutant binding to HA-VP3 were quantified using gray-value analysis and normalized by the relative densities of HA-VP3. The activity of binding of each Flag-VP6 mutant to HA-VP3 was calculated in five experiments, and the results (means ± SDs) are shown as a percentage of the binding of the WT. *, a significant difference in comparison to the binding activity of WT Flag-VP6 (*P* < 0.01). (D) The relative density of HA-VP3 binding to each of the Flag-VP6 mutants was quantified using gray-value analysis and normalized by the relative density of each Flag-VP6 mutant. The activity of HA-VP3 binding to each Flag-VP6 mutant was calculated in five experiments, and the results (means ± SDs) are shown as a percentage of the binding of the WT. *, a significant difference in comparison to the activity of HA-VP3 binding to WT Flag-VP6 (*P* < 0.01).

We further interrogated the C-terminal motif between residues 279 and 286 in order to identify which residues were specifically involved in coordinating the interaction between VP6 and VP3. Here, we introduced point mutations into this 8-residue motif (VVRATAYF) in the Flag-tagged VP6 and analyzed the ability of these mutants to interact with VP3 (Fig. 6A). Among the 8 residues of the binding region, we focused on three particular residues: R281, Y285, and F286. Each of these amino acid residues was individually replaced by alanine in pairs and as a triplet (Fig. 6A). In addition, as residues 279 to 286 likely form a beta-sheet structure, on the basis of the findings of our nuclear magnetic resonance analysis (22), an additional mutant in which 2 further residues, A282 and A284, in the VP3-binding motif were replaced by glycine to partially destroy the predicted beta-sheet structure was tested. Note that the secondary structure prediction of mutated VP6 (determined with the Jpred 4 protein secondary structure prediction server [http://www.compbio.dundee.ac.uk/jpred/]) showed that alanine mutations of R281, Y285, and F286 would not destroy the beta-sheet structure. As a control, a VP6 truncation mutant in which the 8-amino-acid VP3-binding motif was deleted (the d278/287 mutant) was used.

All four Flag-tagged VP6 mutants in which Y285 was replaced by alanine (the RYF/3A, RY/2A, YF/2A, and YA mutants) lost their ability to interact with VP3 (Fig. 6B to D), suggesting that the substitution of Y285 had a profound effect on the interaction of VP6 with VP3. In contrast, the single substitution of R281 for alanine (RA) and the A282/A284 (AA/2G) double mutation did not affect the VP3-binding activity (Fig. 6B to D).

Both VP6 mutants in which F286 was mutated (the FA single mutant and the RF/2A double mutant) showed an intermediate phenotype with substantially reduced but not wholly ablated binding to VP3 (Fig. 6B to D). The FA single mutant retained a greater capacity to bind VP3 than the RF/2A double mutant, suggesting that residues R281 and F286 may be involved in coordinating the interaction of VP6 with VP3.

Furthermore, the sequence of this region of VP6, particularly residues R281, Y285, and F286, appeared to be highly conserved among all BTV serotypes, a finding that supports our data and further implies that these are important functional residues comprising a VP3-binding motif in the C terminus of VP6.

### Disruption of the VP6/VP3 interaction perturbs BTV replication.

To confirm the results of our *in vitro* and biochemical analysis, we further interrogated the interaction between VP6 and VP3 in a live virus system, using our RG system to generate BTV strains with mutations in VP6 at C-terminal positions R281, Y285, and F286. We generated two VP6/VP3 interaction-defective viruses: one in which the 8-amino-acid VP3-binding motif was deleted (BTV d278/287) and one that had a triple-substitution mutation (BTV RYF/3A). Additionally, a BTV strain with a mutant VP6 containing the substitution R281A (BTV RA) was used as a control.

**FIG 7:**
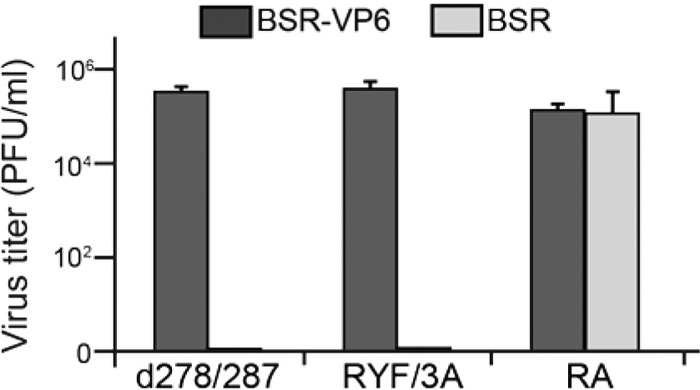
Assay of VP6/VP3 interaction-defective BTV, BTV RYF/3A (RYF/3A), and BTV d278/287 (d278/287) replication. Each 100 μl (∼1 × 10^3^ PFU) of VP6/VP3 interaction-defective BTV once amplified in BSR-VP6 cells was inoculated into either WT-BSR cells (light gray) or BSR-VP6 cells (dark gray). At 24 h postinoculation, the total virus titer (mean ± SD) was determined by plaque assay. As a control, cells were infected with BTV RA (RA).

We then analyzed the viability of these mutant strains in BSR-VP6 cells and found that all three BTV VP6 mutants could replicate in the VP6-complemented cell line (Fig. 7). Subsequently, we infected WT-BSR cells with the mutant viruses recovered from VP6-BSR cells and found that while BTV RA replicated in WT-BSR cells to similar levels as in BSR-VP6 cells, the two VP6/VP3 interaction-defective viruses (BTV d278/287 and BTV RYF/3A) could not replicate in the noncomplementing cell line (Fig. 7).

**FIG 8:**
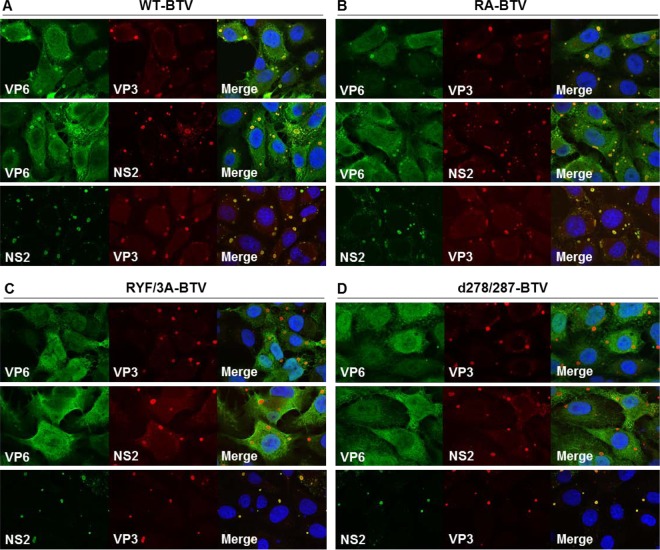
Localization of VP6, VP3, and NS2 in BTV-infected cells. WT-BSR cells were infected with each of WT-BTV (A), RA-BTV (B), RYF/3A-BTV (C), and d278/287-BTV (D), expressing WT VP6, RA VP6, RYF/3A VP6, and d278/287 VP6, respectively. Note that the cells were infected with WT-BTV and RA-BTV at an MOI of 0.1. At 24 h postinfection, the expression of VP6, VP3, and NS2 was observed using confocal microscopy. VP6 was detected using a guinea pig anti-VP6 antibody (top) and a rabbit anti-VP6 antibody (middle). VP3 was detected using a mouse anti-VP3 antibody. NS2 was detected using a guinea pig anti-NS2 antibody.

To further confirm that the two VP6/VP3 interaction-defective mutants (BTV d278/287 and BTV RYF/3A) could not replicate due to disruption of the VP6/VP3 interaction, the localization of mutant VP6, VP3, and NS2 in infected cells was analyzed by confocal microscopy. WT-BSR cells were infected with one of these viruses as well as with the BTV RA mutant, in which the VP6/VP3 interaction was not perturbed. As a control, WT BTV was also used to infect BSR cells. At 24 h postinfection, VP6 proteins of the WT virus and the RA mutant virus were predominantly colocalized with VP3 and NS2 within VIBs (Fig. 8A and B, top and middle). In contrast, in the RYF/3A and d278/287 mutant virus-infected cells, VP6 was diffused and detected throughout the cytosol and in a smaller amount in the VIBs (Fig. 8C and D, top and middle). In contrast, the VP3 protein was clearly detectable within the VIBs, similar to the findings obtained with WT virus infection (Fig. 8A to D, bottom). These results support the suggestion that VP6 interacts with VP3 via these two motifs in the VIBs, where the primary replicase complex assembles.

**FIG 9:**
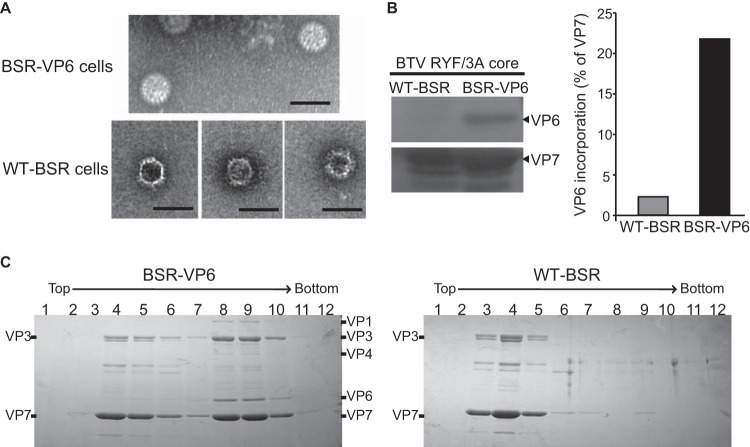
Nonrecruitment of VP6/VP3 interaction-defective VP6 as well as VP1, VP4, and RNAs into core particles purified from WT-BSR cells. (A) Electron microscopy of BTV RYF/3A core particles amplified in either BSR-VP6 cells (top) or normal BSR cells (bottom). Bars, 100 nm. (B) The incorporation of VP6 proteins with core particles was analyzed using immunoblotting (left). VP6 proteins were normalized with VP7, and the level of incorporation with VP6 into the particles is shown as a percentage of that for VP7 proteins (right). (C) Analysis of VP6/VP3 interaction-defective BTV core particles purified from WT-BSR and BSR-VP6 cells by CsCl gradient centrifugation. Supernatants from BSR-VP6 (left) or BSR (right) infected cell lysates were spun down over a 30% (wt/vol) sucrose cushion and subjected to CsCl equilibrium centrifugation. Thirteen fractions were collected from the top, and then 12 fractions from the top were analyzed by SDS-PAGE. The gels were stained with Coomassie brilliant blue.

To elucidate the effect of the interaction between VP6 and VP3 in core assembly, both WT-BSR and BSR-VP6 cells were infected with the BTV RYF/3A mutant. At 3 days postinfection, core particles were purified by a sucrose ultracentrifugation method. Electron microscopy analysis showed that the majority of core particles purified from WT-BSR cells were empty (Fig. 9A, bottom), whereas particles purified from BSR-VP6 cells were complete (Fig. 9A, top). These data indicate a role for VP6 in the formation of the core, potentially including a role in genome packaging.

Our previous study demonstrated that defective VP6 is not incorporated within BTV particles (4). To assess whether mutation of the three residues in VP6 (R281, Y285, and F286) responsible for the VP6/VP3 interaction could affect the incorporation of VP6 within the capsid, core particles obtained from WT-BSR and BSR-VP6 cells were further analyzed using immunoblotting. The VP6 and VP7 proteins were detected using anti-VP6 and anti-VP7 antibodies, respectively. Small amounts of VP6 protein were detected in core particles that were semipurified from infected WT-BSR cells; in contrast, a larger amount of the VP6 protein was detected in core particles obtained from infected BSR-VP6 cells (Fig. 9B, left). The amount of VP7 protein was the same in both samples (Fig. 9B, left). To further confirm that the RYF/3A VP6 was unable to incorporate within core particles, the presence of the VP6 protein in core particles was quantified by normalizing the amount of VP6 protein to the amount of VP7 (Fig. 9B, right). The amount of VP6 incorporated into core particles semipurified from infected WT-BSR cells was clearly smaller than that from infected BSR-VP6 cells, suggesting that few core particles purified from WT-BSR cells contained the VP6 protein, and these could be residual core particles of the inoculated virus obtained from BSR-VP6 cells with WT VP6 but not RYF/3A VP6.

To further confirm this, BTV RYF/3A core particles semipurified from WT-BSR and BSR-VP6 cells were further analyzed using a cesium chloride (CsCl) equilibrium centrifugation as described in Materials and Methods. Core particles produced in BSR-VP6 cells showed two peaks: one was with VP6 (predominantly in fractions 8 and 9), and the other was without VP6 (predominantly in fractions 4 and 5) (Fig. 9C, left). Although a very small amount of VP6 was detected in fractions 4 to 7, the amount of VP6 protein was the same in all four fractions and did not correlate with the peak of VP7 and VP3 (Fig. 9C, left), suggesting that the VP6 proteins did not interact with VP7 or VP3. In contrast, core particles produced in WT-BSR cells mainly showed one peak (predominantly in fraction 4), which did not contain VP6 (Fig. 9C, right). Very small amounts of core proteins were detected in fraction 9 of the WT-BSR cell sample, which could be remaining core particles of the inoculated virus (Fig. 9C, right). Neither VP1 nor VP4 was detected in fractions 3 to 5 of the WT-BSR cell sample or fractions 4 to 6 of the BSR-VP6 cell sample, suggesting that without VP6, VP1 and VP4 cannot be recruited into the particles. These results clearly demonstrate that the disruption of the VP6/VP3 interaction caused the failure of recruitment of the transcription complex (VP1/VP4/VP6/ssRNA) into the core particle.

In summary, here we have presented data which demonstrate that orbivirus core protein VP6 is translocated to VIBs during virus replication and interacts with VP3, the subcore protein. Furthermore, we have demonstrated that residues R281, Y285, and F286 in the VP6 C terminus are responsible for directing the interaction of VP6 with VP3. The interaction between VP6 and VP3 plays a key role during virus replication, specifically, in assembly of the virus core, and may include a role in genome packaging.

## DISCUSSION

Virus particle assembly and genome packaging are complex and highly dynamic processes. These processes are interlinked and are dependent on multiple synchronized and sequential protein-protein and protein-nucleic acid interactions. The mechanisms by which these pathways proceed and the interactions formed therein are not fully understood for many virus families, including the segmented dsRNA viruses of the Reoviridae, such as orthoreovirus, rotavirus (RV), and BTV. Reoviridae is a family of highly complex viruses for which multiple structural proteins (e.g., seven in BTV) must be coordinated to form a doubled-layered viral core particle, which subsequently acquires other capsid proteins to generate infectious, mature virion particles. During core formation, multiple genome segments (e.g., 10 in BTV) must be packaged as a complete set and in a formation which permits replication in subsequent host cells. The exact mechanism by which this is achieved is largely unknown.

We have previously shown that BTV (and other members of the Orbivirus genus) differ from other members of the Reoviridae family, as they possess an additional unique inner capsid protein, VP6. The VP6 protein has an essential role in virus assembly and is a component of the primary replicase complex (subcore) (2–4). However, the exact location of VP6 in the core, the mechanism by which it is recruited, and the functional roles that it has in virus replication are not well defined. Here, we demonstrate for the first time that VP6 interacts directly with the virus inner core protein VP3 and that this interaction appears to be important for the recruitment of VP6 into the VIBs, which are the virus-induced sites of virus replication and assembly in the cell. Furthermore, our data suggest that the recruitment of VP6 into the VIBs may be an important early step in coordinating the packaging of the RNA genome into virus core particles.

The assembly of BTV cores initiates with the assembly of a subcore particle, which includes minor enzymatic structural proteins VP1, VP4, and VP6 alongside the 10 genomic RNAs, and the subsequent addition of VP3 decamers (4, 17, 18, 23). It has previously been demonstrated that the *in vitro* expression of VP1 and VP4 results in the formation of a protein complex which is recruited into core-like particles when it is coexpressed together with VP3 and VP7 (24–26). However, neither VP1 nor VP4 was incorporated into core particles in the absence of VP6 in an RG-based live virus model (4), suggesting that the incorporation of VP6 is important in core formation and that this interaction may depend on VP3. In the current study, we demonstrated that VP6, which has 328 amino acid residues, interacts directly with VP3 via its C-terminal region and that this interaction may play a critical role in genome packaging. Similar interactions have been reported for other viruses (27–34). For example, the Ebola virus nucleoprotein (NP) and matrix protein VP40 interact through the C-terminal region of NP (28, 31, 32), and the interaction of influenza A virus NP and matrix protein is important for genome incorporation (27, 33).

The importance of the capsid protein for genome packaging is also suggested in the other members of the family Reoviridae, which do not have a BTV VP6-like protein (35). Inner capsid (core) particles of RV consist of only two enzymatic proteins, VP1 (polymerase) and VP3 (capping enzyme), and an inner capsid protein, VP2 (36–38). VP1 and VP3 are believed to form a viral polymerase complex with viral dsRNAs and to be attached to the inner side of the VP2 shell (36–38). Although no direct evidence of the involvement of VP2 during RV genome packaging is available, deletion of the N-terminal region of RV VP2 resulted in the failure of VP3 and VP1 to be taken up into the VP2 shell (39). As RV VP1 is an RNA-dependent RNA polymerase which should bind to the 3′ end of RV positive-strand RNAs (40–42), it is possible that the interaction of VP1 with VP2 is important to genome packaging. In the case of RV, instead of the BTV VP6-like protein, two nonstructural proteins (NSP2 and NSP5) which are involved in viroplasm formation may be involved in genome packaging (35, 43, 44).

During orbivirus replication, the phosphorylated NS2 is the initiator of the formation of VIBs in virus-infected cells (13, 16, 23). The components of the BTV core (VP1, VP4, VP3, VP6, and genomic RNA) are recruited into VIBs, where core assembly takes place (13–16). Our data presented here indicate that the translocation of EGFP-VP6 (VP6/N_87_C_115_) into VIBs is led by VP3 and that VP6 and VP3 interact via the VP6 C-terminal region, specifically, residues 279 to 286 of the total 328 residues. We did not observe a direct interaction between VP6 and NS2, in contrast to a previous report of a direct interaction between VP6 and NS2 (13–16). This discrepancy may be due to the fact that VP6 interacts with NS2 via an unknown region from residues 88 to 214, which were deleted in our current study. Alternatively, since VP6 is likely to be recruited into VIBs early in orbivirus replication (at 7 h postinfection) (45), it may also be possible that the efficient translocation of VP6 into VIBs early in virus replication is directed by VP3. In addition, our data obtained using the VP6/VP3 interaction-defective BTV mutants RYF/3A and d278/287 showed that defective VP6 proteins could only partially translocate into VIBs and were visualized with both VP3 and NS2 at the edge of VIBs. The recruitment of core components, including the viral genome, into VIBs should be directed not only by the VP6/VP3 interaction but also by other interactions among components, as well as host factors. Thus, although the temporal sequence of viral protein and RNA recruitment into VIBs requires further investigation, it is clear that the VP6/VP3 interaction is involved in the recruitment of VP6 into the VIBs.

Further, using a series of *in vitro* and *in vivo* methods, we demonstrated that the binding of VP6 to VP3 is highly specific and requires a specific 8-residue motif (_279_VVRATAYF_286_), a VP6 motif shared by other BTV serotypes. We particularly focused on understanding the role of three residues of this motif, arginine 281, tyrosine 285, and phenylalanine 286. Aromatic residues are key components of a protein's structure and are important for stability as well as protein-protein interactions. In addition, the hydrophobic core formed by bulky amino acids is essential for the functions of proteins (46–48). We found that both tyrosine 285 and phenylalanine 286 are involved in the VP3 interaction. Although the precise structure and location of VP6 in core particles have not been resolved, these aromatic residues may be involved in stacking interactions with each other or with other aromatic side chains of VP3. Arginine 281 did not affect the VP3 interaction by itself; however, together with an aromatic residue, phenylalanine 286, the residue contributed to the interaction. A cationic side chain and an aromatic side chain nearby are involved in the cation-pi interaction, which is an important noncovalent binding interaction in the protein structure (49–52). Although further studies will be necessary to determine the influence of tyrosine 285 and phenylalanine 286 in virus replication, all three residues, arginine 281, tyrosine 285, and phenylalanine 286, are possibly important for retention of the conformation of the VP3-binding site in VP6.

According to data from our previous study (4), CsCl equilibrium centrifugation analysis of core particles purified from BSR-VP6 cells infected with a mutated BTV which expressed defective EGFP-VP6 showed that there was only one peak of core particles with WT VP6. However, our current data obtained using BTV RYF/3A showed that there were two peaks; one of them contained complete core particles with VP6 proteins, and the other contained empty particles lacking the VP6 protein. RYF/3A VP6 proteins expressed in BSR-VP6 cells likely interrupt the incorporation of WT VP6 into core particles. Since core particles obtained from BTV RYF/3A-infected WT-BSR cells did not contain VP6 proteins, RYF/3A VP6 was not incorporated into core particles without the aid of VP3. As a previous study suggested that VP6 is an oligomeric protein (21), it is possible that WT and defective VP6 proteins may still form oligomers but may not be functional. As the sizes of the WT and defective VP6 proteins were the same, it is impossible to define whether the VP6 proteins, which were incorporated into core particles that were purified from infected BSR-VP6 cells, were only WT proteins or heterooligomers of WT and defective proteins. Further studies, including structural studies, would be required to better understand whether RYF/3A VP6 loses only VP3-binding activity but retains other functions and whether the mutated protein could compete with WT proteins to reduce the yield of complete core particles even in BSR-VP6 cells.

The disruption of the VP6/VP3 interaction by mutating 3 amino acid residues at the putative VP3-binding site in the VP6 protein critically affects both the incorporation of VP6 within the particle and the recruitment of the other two inner capsid proteins, VP1 and VP4, as well as virus RNAs. Our current and previous data (Fig. 8) (2–4) demonstrate that the BTV proteins, including mutated VP6, are expressed in WT-BSR cells infected with defective BTV mutants, which suggests that mRNA synthesis from these BTV core particles is likely to proceed as it does in WT BTV infection. Thus, it is clear that the packaging inefficiency of BTV RYF/3A is not due to a decrease in the amount of RNA or mutated VP6 protein but is due to the defective interaction of VP6 with VP3.

In summary, we present here the first evidence that BTV VP6 interacts directly with the inner core protein VP3 and that this interaction is dependent on an 8-amino-acid motif in the VP6 C-terminal region. Moreover, we obtained data that clearly indicate that aromatic residues within this region are likely to be the key residues in coordinating this interaction. We generated data from *in vitro* protein expression experiments, biochemical analyses, and *in vivo* experiments by direct mutagenesis of the replicating viral genome which supported the suggestion that the VP3-VP6 interaction is an important early event in viable core formation and is probably necessary in directing genome packaging. However, future studies are needed to define the precise location of VP6 in the virus core and to further our understanding of the role of VP6 in virus assembly and genome packaging.

## MATERIALS AND METHODS

### Cell lines.

Wild-type BSR cells (WT-BSR cells; BSR cells are subclones of BHK-21 cell) were maintained in Dulbecco's modified Eagle's medium (DMEM; Sigma) supplemented with 4% (vol/vol) fetal bovine serum (FBS; Thermo Fisher Scientific). WT-BSR cells modified to stably express BTV VP6 (BSR-VP6) (3) were maintained in DMEM supplemented with 4% (vol/vol) FBS and 7.5 μg/ml of puromycin (InvivoGen).

Two Spodoptera frugiperda-derived cell lines were used: Sf9 cells were maintained in Sf900II medium (Thermo Fisher Scientific), and Sf21 cells were maintained in TC-100 medium (Sigma) supplemented with 10% (vol/vol) FBS.

### Plasmids and BTV T7 transcripts.

For construction of recombinant baculoviruses expressing BTV VP6 and VP3, the coding regions (CDR) of BTV-1 S3 and BTV-10 S9 (encoding VP3 and VP6, respectively) were inserted into the transfer vector pFastBac1 (Thermo Fisher Scientific). To generate transfer vectors for recombinant baculoviruses expressing N-terminal tagged proteins, each of the cDNAs encoding the HA tag (YPYDVPDYA) and the His tag (HHHHHH), together with a spacer and the HRV3C protease recognition sequence (SSG-LEVLFQGP), was inserted in frame into the 5′ end of either VP3 or VP6 cDNA.

Two mammalian expression plasmids were generated: pCAG-PBTV1-HAVP3, expressing an N-terminal HA-tagged VP3 (HA-VP3), which was inserted into pCAG-PM (58), and pcDNA-BTV10-FlagVP6, expressing a Flag (YKDDDDK)-tagged VP6 (Flag-VP6), which was inserted into pcDNA3.1 (Clontech).

Mammalian expression plasmids for the RG system, pCAG-PBTV1VP1, pCAG-PBTV1VP3, pCAG-PBTV1VP4, pCAG-PBTV10VP6, pCAG-PBTV1VP7, and pCAG-PBTV1NS2, were described previously (3, 4, 22). T7 plasmids for the BTV transcripts used in the RG system were described previously (3, 53).

Segment S9 (BTV-10) was modified by site-directed mutagenesis, using a method described previously (54), to generate a series of EGFP-BTV mutants from a T7 plasmid, S9-EGFP277/656, which was described previously (3) and which includes 276 nucleotides from the segment S9 5′ end and 393 nucleotides from the segment S9 3′ end, which should retain segment packaging signals.

Since an inadvertent loss of an adenine nucleotide from the EGFP stop codon (TAA → TA-) was found in S9-EGFP277/656 when the virus stock was obtained (unpublished data), a stop codon was changed (TAA → TAG). The guanine of the stop codon at the end of the EGFP gene was then deleted. The deletion spontaneously created a tyrosine (TAG → TAC^657^) residue instead of a stop codon by shifting the remaining nucleotides (nucleotides [nt] 657 to 1049) of S9 to sequentially synthesize a C-terminal region (residues 215 to 329, nt 658 to 1005) of VP6 in frame. Further truncations were generated on the basis of these two S9-EGFP T7 plasmids. To generate mammalian expression plasmids for chimeric EGFP-VP6 mutants, each CDR of T7 plasmids for the S9-EGFP mutants was inserted into pCAG-PM. The sequence of each modified VP6 and S9 plasmid was confirmed.

For the synthesis of uncapped T7 transcripts, a RiboMAX large-scale RNA production system—T7 (Promega) was used according to the manufacturer's protocols, as described previously (3).

### Recovery of BTV with mutated VP6.

Confluent monolayers of BSR-VP6 cells were first transfected with 6 mammalian expression plasmids encoding VP1, VP3, VP4, VP6, VP7, and NS2, followed by a second transfection with uncapped BTV T7 transcripts, using the Lipofectamine 2000 reagent (Thermo Fisher Scientific), as described previously (4, 22). At 6 h after the second transfection, the culture medium was replaced with fresh DMEM supplemented with 5% FBS, and transfected cells were incubated at 35°C in 5% CO_2_ for 3 days and monitored for the appearance of a cytopathic effect (CPE). Note that BTV-10 (U.S. isolate) genome segment 9 was used in this study.

BTV stocks with mutated VP6 were obtained by infecting BSR-VP6 cells at a low multiplicity of infection (MOI) and harvested when a 90 to 100% CPE was evident. Note that all mutant BTV strains were kept at a low passage number (less than 5) for all experiments. The profiles of dsRNA, especially the sequences of the mutated S9 segment, extracted from BTV with mutated VP6 were confirmed, and the virus stocks were stored at 4°C.

### Generation of recombinant baculoviruses.

Recombinant baculovirus constructs were prepared with the transfer vectors described above using a Bac-to-Bac baculovirus expression system (Thermo Fisher Scientific) according to the manufacturer's procedures, producing five virus constructs: Ac/BTV10/His-VP6, Ac/BTV10/VP6, Ac/BTV1/His-VP3, Ac/BTV1/HA-VP3, and Ac/BTV1/VP3. Briefly, competent Escherichia coli DH10Bac cells (Thermo Fisher Scientific) were transformed with each of the transfer vectors and selected according to the manufacturer's procedures. Confluent monolayers of Sf21 cells were transfected with miniprep bacmid DNA extracted from a transformed DH10Bac clone using the Insect GeneJuice transfer reagent (Merck Millipore). At 24 h posttransfection, the culture medium was replaced with fresh TC-100 medium containing 10% FBS and the cells were incubated at 27°C for 3 days until a CPE appeared. Culture supernatants were collected and inoculated into Sf9 cells. At 3 days postinoculation, the culture supernatants and cells were harvested separately and the expression of proteins was confirmed by SDS-PAGE and immunoblotting using standard methods. Virus stocks were prepared by infecting Sf9 cells at an MOI of 0.01 and harvested at 4 days postinfection. All stocks were stored at 4°C.

### Coexpression and copurification of BTV VP6 and VP3.

For the coexpression of VP6 and VP3, three sets of recombinant baculovirus combinations were used. To copurify VP6 with VP3, nickel affinity chromatography was used as described previously, but with some modifications (22, 55). Briefly, Sf9 cells were infected with Ac/BTV10/His-VP6 at an MOI of 2.5 together with either Ac/BTV1/HA-VP3 or Ac/BTV1/VP3 at an MOI of 5.0. In parallel, Sf9 cells were infected with Ac/BTV1/His-VP3 (MOI, 5.0) together with Ac/BTV10/VP6 (MOI, 2.5). At 2 days postinfection, the cells were harvested and lysed with VP3 lysis buffer containing 50 mM Tris-HCl (pH 8.0), 200 mM NaCl, 1.0% (wt/vol) Triton X-100, and protease inhibitor cocktail (Nacalai Tesque). Nonnuclear RNA and DNA were removed by treatment with 10 μg/ml of RNase A (Nippon Gene) and 0.3 U/ml of DNase (Nippon Gene) at 37°C for 15 min. After removal of the nuclei and cell debris, the cell lysate was mixed with Cosmogel His-Accept resin (Nacalai Tesque) for 1 h at 4°C. After the affinity gel was washed with 50 mM sodium phosphate buffer (pH 8.0) containing 10% glycerol, 200 mM NaCl, and 20 mM imidazole, His-tagged proteins and interacted proteins were eluted with 50 mM sodium phosphate buffer (pH 8.0) containing 10% glycerol, 200 mM NaCl, and 250 mM imidazole. Fractions of 1.0 ml each were collected and analyzed by SDS-PAGE.

### Size exclusion chromatography.

The complex of VP6 and VP3 was analyzed by use of a HiPrep 16/60 Sephacryl S-300 HR column (GE Healthcare) in a buffer containing 20 mM Tris-HCl (pH 8.0) and 100 mM NaCl. Note that the VP3 protein was easily aggregated in the presence of a low concentration of NaCl (less than 100 mM). The flow rate was 0.5 ml/min, and 3.0-ml fractions were collected and analyzed by SDS-PAGE.

### Immunoprecipitation and immunoblotting.

Each Flag-VP6 protein or a series of mutated Flag-VP6 proteins was coexpressed with HA-VP3 in WT-BSR cells. Briefly, 2 wells of BSR cells (90% confluence) in a 6-well plate (Sigma) were transfected with 1.5 μg/well of a plasmid for Flag-VP6 or a series of mutated Flag-VP6 proteins and 1.5 μg/well of pCAG-BTV1-HAVP3 using the X-tremeGENE HP transfection reagent (Roche). At 24 h posttransfection, the cells were lysed with 500 μl/well of lysis buffer containing 100 mM Tris-HCl (pH 7.4), 100 mM NaCl, 1 mM EDTA, and 1.0% (wt/vol) Triton X-100. Nuclei and cell debris were removed by centrifugation at 20,000 × *g* for 5 min at 4°C. The soluble fraction of the cell lysate was mixed with either 1.0 μg of a mouse anti-HA tag monoclonal antibody (MAb; MBL) or an anti-DDDDK tag MAb (MBL) for 16 h at 4°C and then mixed with Cosmogel Ig-Accept protein G resin (Nacalai Tesque) for 2 h at 4°C. After unbound proteins were removed by washing the resins with VP3 lysis buffer, intact proteins were collected from the resins with 1× SDS-PAGE sample buffer containing 50 mM Tris-HCl (pH 6.8), 1.0% (vol/wt) SDS, 10% (vol/vol) glycerol, 0.1% (wt/vol) bromophenol blue, and 0.1 M dithiothreitol and analyzed by immunoblotting. Flag-VP6 and HA-VP3 were detected using a rabbit anti-DDDDK-tagged polyclonal antibody (pAb; MBL) and a rabbit anti-HA-tagged pAb (MBL), respectively. Proteins were visualized using alkaline phosphatase-conjugated goat anti-rabbit immunoglobulin G (IgG; Sigma) and substrates for alkaline phosphatase (Promega). Each detected band was quantified using CS Analyzer (version 4) software (Atto). The activities of Flag-VP6 binding to HA-VP3 were normalized by the activities of HA-VP3 binding to the anti-HA MAb. In the same way, the activities of HA-VP3 binding to Flag-VP6 were normalized by the activities of Flag-VP6 binding to the anti-Flag MAb. For detection of BTV proteins, standard methods of immunoblotting were used. To detect VP6 and VP7, an anti-VP6 pAb produced in a rabbit and an anti-VP7 pAb produced in a rabbit were used, respectively. The incorporation of the VP6 protein into core particles was shown by normalization of the amount of VP6 to the amount of the VP7 protein.

### Immunofluorescence.

WT-BSR cells were either infected with BTV mutated VP6 at an MOI of 1.0 or transfected with several combinations of mammalian expression plasmids for mutant proteins, pCAG-PBTV1VP3, pCAG-PBTV1NS2, and pCAG-PBTV10VP6-EGFP. At 24 h postinfection or posttransfection, the cells were washed once with phosphate-buffered saline (PBS) and fixed in 4.0% (wt/vol) paraformaldehyde in PBS. The cells were permeabilized in 0.2% (vol/vol) Triton X-100 in PBS. Staining was performed using a mouse anti-VP3 pAb, a guinea pig anti-NS2 pAb, a guinea pig anti-VP6 pAb, and a rabbit anti-VP6 pAb, followed by Alexa Fluor 564- or Alexa Fluor 488-conjugated goat anti-mouse IgG (Thermo Fisher Scientific), Alexa Fluor 564- or Alexa Fluor 488-conjugated goat anti-guinea pig IgG (Thermo Fisher Scientific), and Alexa Fluor 488-conjugated goat anti-rabbit IgG (Thermo Fisher Scientific). Nuclei were stained with 4′,6-diamidino-2-phenylindole (DAPI; Nacalai Tesque) using a standard protocol. Fluorescence was observed using fluorescence microscopy (with a model BZ-9000 microscope; Keyence) and confocal microscopy (with a model LSM 510 microscope; Zeiss).

### BTV replication assay.

For calculation of the virus titer over the passages, each 100 μl of BTV mutated VP6 recovered from transfected BSR-VP6 cells was amplified once in BSR-VP6 cells. The supernatant and cells were collected at 2 days postinfection (passage 0 [P0]), and 100 μl of each P0 supernatant was inoculated into WT-BSR cells and BSR-VP6 cells. At 24 h postinfection, each supernatant and the cells were collected (P1). The virus titer of each collected supernatant (P1) was determined by plaque assay using BSR-VP6 cells.

### Purification of BTV core particles and electron microscopy.

BTV core particles were semipurified from WT-BSR cells and BSR-VP6 cells as described previously, with modifications (56, 57). Briefly, infected cells were lysed at 72 h postinfection with lysis buffer containing 50 mM Tris-HCl (pH 7.4), 150 mM NaCl, 2 mM EDTA (pH 8.0), and 0.1% (wt/vol) NP-40. After the nuclei and cell debris were removed, α-chymotrypsin and *N*-lauroyl sarcosine (sodium salt) were added to 60 μg/ml and 0.1% (wt/vol), respectively, and the samples were incubated at 35°C for 1 h. Ultracentrifugation on a sucrose cushion consisting of 30% (wt/vol) sucrose and 20 mM Tris-HCl (pH 8.0) was carried out at 96,281 × *g* (average) and 4°C for 1.5 h. The pellet was resuspended in 20 mM Tris-HCl (pH 8.0). Aliquots of semipurified cores were absorbed onto Formvar/carbon support film copper 200-mesh grids (Nisshin EM) for 1 min, washed with water, and negatively stained with 2% (wt/vol) uranyl acetate. The grids were examined under an electron microscope (model JEM-1400 microscope; JEOL).

Further purification of core particles by CsCl equilibrium centrifugation was performed using a modification of the previously described methods (4). Briefly, CsCl, which was diluted with 20 mM Tris-HCl (pH 7.4) up to 5.0 ml to reach a final concentration of 50% (wt/vol), was added to the semipurified core particles. The mixture was centrifuged at 142,743 × *g* (average) for 18 h at 16°C. After centrifugation, 13 fractions (400 μl each) were collected from the top and pelleted through a 30% (wt/vol) sucrose cushion by centrifugation at 20,000 × *g* for 1.5 h at 4°C. The pellets were resuspended in 20 mM Tris-HCl (pH 8.0) and analyzed by SDS-PAGE.
